# Exogenous GABA Enhances Copper Stress Resilience in Rice Plants via Antioxidant Defense Mechanisms, Gene Regulation, Mineral Uptake, and Copper Homeostasis

**DOI:** 10.3390/antiox13060700

**Published:** 2024-06-07

**Authors:** Zakirullah Khan, Rahmatullah Jan, Saleem Asif, Muhammad Farooq, Kyung-Min Kim

**Affiliations:** 1 Department of Applied Biosciences, Graduate School, Kyungpook National University, Daegu 41566, Republic of Korea; zakir371@knu.ac.kr (Z.K.); saleemasif10@knu.ac.kr (S.A.); 2018225016@knu.ac.kr (M.F.); 2 Coastal Agriculture Research Institute, Kyungpook National University, Daegu 41566, Republic of Korea

**Keywords:** copper, gamma aminobutyric acid, oxidative stress, antioxidant enzymes, genes expression

## Abstract

The importance of gamma-aminobutyric acid (GABA) in plants has been highlighted due to its critical role in mitigating metal toxicity, specifically countering the inhibitory effects of copper stress on rice plants. This study involved pre-treating rice plants with 1 mM GABA for one week, followed by exposure to varying concentrations of copper at 50 μM, 100 μM, and 200 μM. Under copper stress, particularly at 100 μM and 200 μM, plant height, biomass, chlorophyll content, relative water content, mineral content, and antioxidant activity decreased significantly compared to control conditions. However, GABA treatment significantly alleviated the adverse effects of copper stress. It increased plant height by 13%, 18%, and 32%; plant biomass by 28%, 52%, and 60%; chlorophyll content by 12%, 30%, and 24%; and relative water content by 10%, 24%, and 26% in comparison to the C50, C100, and C200 treatments. Furthermore, GABA treatment effectively reduced electrolyte leakage by 11%, 34%, and 39%, and the concentration of reactive oxygen species, such as malondialdehyde (MDA), by 9%, 22%, and 27%, hydrogen peroxide (H_2_O_2_) by 12%, 38%, and 30%, and superoxide anion content by 8%, 33, and 39% in comparison to C50, C100, and C200 treatments. Additionally, GABA supplementation led to elevated levels of glutathione by 69% and 80%, superoxide dismutase by 22% and 125%, ascorbate peroxidase by 12% and 125%, and catalase by 75% and 100% in the C100+G and C200+G groups as compared to the C100 and C200 treatments. Similarly, GABA application upregulated the expression of GABA shunt pathway-related genes, including gamma-aminobutyric transaminase (*OsGABA-T*) by 38% and 80% and succinic semialdehyde dehydrogenase (*OsSSADH*) by 60% and 94% in the C100+G and C200+G groups, respectively, as compared to the C100 and C200 treatments. Conversely, the expression of gamma-aminobutyric acid dehydrogenase (*OsGAD*) was downregulated. GABA application reduced the absorption of Cu^2+^ by 54% and 47% in C100+G and C200+G groups as compared to C100, and C200 treatments. Moreover, GABA treatment enhanced the uptake of Ca^2+^ by 26% and 82%, Mg^2+^ by 12% and 67%, and K^+^ by 28% and 128% in the C100+G and C200+G groups as compared to C100, and C200 treatments. These findings underscore the pivotal role of GABA-induced enhancements in various physiological and molecular processes, such as plant growth, chlorophyll content, water content, antioxidant capacity, gene regulation, mineral uptake, and copper sequestration, in enhancing plant tolerance to copper stress. Such mechanistic insights offer promising implications for the advancement of safe and sustainable food production practices.

## 1. Introduction

Rice (*Oryza sativa*) stands as the primary staple and cereal, sustaining two-thirds of the world’s population [1]. Its nutritional richness and high energy value make it a cornerstone of human sustenance, supporting livelihoods across diverse communities [2]. Rice holds significant importance in Asian populations, both in production and consumption. It accounts for up to 50% of the dietary caloric supply and contributes substantially to protein intake for approximately 520 million people living in poverty across Asia [3]. Additionally, rice serves as the main source of income and employment for over 200 million households across various developing nations, further highlighting its economic significance and role in sustaining livelihoods [4]. Human activities like industrialization, extensive mining, and already established agricultural practices are causing a continuous buildup of heavy metals [5], including cadmium, chromium, lead, mercury, copper, and nickel, within our agricultural systems [6].

Among these metals, copper is particularly noteworthy. While essential as a micronutrient for plant growth, it also presents a dual challenge due to its potentially harmful effects [7]. Its indispensability in the structural and catalytic aspects of numerous proteins and enzymes profoundly supports plant growth and confers stress tolerance [8]. Functionally, copper orchestrates critical processes like mitochondrial respiration, electron transport chain functionality, photosynthesis, cell wall metabolism, lignin synthesis [9], and plays pivotal roles in responding to oxidative stress and facilitating hormone signaling [10]. It serves as a co-factor in vital plant enzymes such as cytochrome c and plastocyanin, playing integral roles in photosynthesis, respiration, nitrogen assimilation, and leaf senescence [11]. However, surpassing optimal levels of copper can precipitate detrimental impacts on both plant physiological and cellular functions, impeding water and nutrient absorption [12]. High concentrations of copper pose toxicity to plant systems, evident through chlorosis, necrosis, stunted growth, and compromised root development [13]. Excessive copper ion interaction with protein sulfhydryl groups can lead to enzymatic inactivation, triggering the formation of reactive oxygen species (ROS) through reactions like Fenton or Haber–Weiss, culminating in oxidative stress [14]. This redox-active metal’s propensity to generate free radicals and other highly reactive ROS significantly damages essential biological molecules and disrupts lipid membranes [15]. Copper accumulation in agricultural fields, especially in rice paddies, is primarily due to the widespread use of copper-based fungicides [16]. These fungicides, essential for controlling various fungal diseases, are applied repeatedly, leading to residual copper in the soil [17]. Paddy fields are particularly vulnerable because the flooded conditions enhance copper solubility and mobility, increasing its uptake by rice plants [18]. Additionally, irrigation with contaminated water and the application of copper-rich fertilizers contribute to this accumulation [19]. Consequently, the long-term use of these inputs results in elevated copper levels in the soil, which can adversely affect soil health and crop productivity [20].

To enhance abiotic stress tolerance, researchers commonly utilize exogenous protectants such as plant hormones, organic acids, signaling molecules, and trace elements [21]. Gamma Aminobutyric Acid (GABA), a ubiquitous non-protein amino acid initially discovered in potato tubers, emerges as an essential endogenous plant signaling molecule pivotal in growth and development, rapidly accumulating in plant tissues during both abiotic and biotic stresses [22,23]. Its application on various crops, including lentils, melons, rice, and wheat, consistently demonstrates its efficacy in mitigating growth inhibition resulting from adverse environmental conditions such as extreme temperatures, drought, salt, light, and oxygen stress [24,25,26,27]. Similarly, GABA has a key role in enhancing tolerance to drought [28], salinity [29], high temperature [24], heavy metals [30], low light intensity, and nitrogen deficiency [31]. GABA promotes plant growth and mitigates stress via up-regulating the antioxidant defense system [32]. Exogenous application of GABA induces growth parameters, promotes antioxidant activity, reduces ROS accumulation, and upregulates GABA shunt genes during As(III) stress [33].

GABA applied exogenously improves amino acid contents, reduces cadmium (Cd^2+^) uptake, and improves GABA-related gene expression [34]. Research has demonstrated that glutamate decarboxylase (*GAD*) stands out as the most responsive gene associated with GABA metabolism when it comes to reacting to abiotic stress [35]. The activity of the *GAD* enzyme and the expression of its associated genes are intricately linked to the improvement of plant stress resistance through GABA mediation [36]. GABA increases the expression of *GABA-T* and *SSADH*, maintaining the fluency of the GABA shunt and TCA metabolism [37]. *GABA-T* and *SSADH* showed significant up-regulation during abiotic stress conditions [38,39].

While recent studies have shown the efficacy of exogenously applied GABA in alleviating the effects of chromium, aluminum, and arsenic stress [30,40,41], its role in responding to copper stress in rice remains an unexplored domain. Therefore, this study seeks to investigate the physiological effects of GABA on copper accumulation, the ensuing biochemical and physiological responses in plants, including antioxidant defense mechanisms. This study aims to elucidate the efficacy of exogenously applied GABA in counteracting copper-induced stress. As far as current knowledge extends, this study marks the pioneering investigation into the role of GABA in addressing copper toxicity in rice.

## 2. Materials and Methods

### 2.1. Plant Material and Growth Conditions

Seeds of the Ilmi rice cultivar (*Oryza sativa* L.) underwent sterilization using Sportak fungicides [42] and multiple rinses with sterilized distilled water. They were soaked in water for four days at 32 °C in an incubator, as per Jan, R., et al. [43]. Pre-germinated seeds were then planted in a specialized soil mix (Doobaena plus) consisting of cocopeat (27%), peat moss (10%), vermiculite (34%), Masato (10%), diatomite (13%), bara mesh (5.5%), fertilizer (0.48%), and humectant (0.2%), provided by Nongkyung Co. Ltd. (Yeongcheon-si, Republic of Korea) [44]. Eight groups of rice plants, five plants per group, were established and grown for 10 days: control only treated with water (CK), 50 μM copper treated (C50), 50 μM copper and GABA treated (C50+G), 100 μM copper treated (C100), 100 μM copper and GABA treated (C100+G), 200 μM copper treated (C200), 200 μM copper and GABA treated (C200+G), and GABA treated (G) plants. Then, the plants were treated with 1 mM GABA at a 2-day interval for a week following prior screening (Supplementary Materials). After one week of GABA treatment, the plants were treated with copper by watering them thoroughly with CuSO_4_ solutions at concentrations of 50 μM, 100 μM, and 200 μM, corresponding to different plant groups. To analyze gene expression, samples were collected randomly after 48 h of Cu treatment, while the rest of the physiological and biochemical analyses were carried out after one month of Cu treatment.

### 2.2. Growth Parameter Evaluation

One month after applying the stress, plant growth including shoot length, root length, and fresh weight were measured, while for the assessment of dry weight (DW) of seedlings, the whole plant including roots and shoots, underwent oven drying at 200 °C for 30 min, followed by maintenance at 60 °C for 48 h, and then weight was calculated [44].

### 2.3. Chlorophyll and Relative Water Contents Measurement

Chlorophyll contents were assessed using a portable chlorophyll meter (SPAD 502, Konica Minolta, Tokyo, Japan). The chlorophyll measurement was performed on the second-to-last fully mature leaf, and readings were taken at three different positions along the leaf: near the leaf base, in the middle, and close to the leaf tip. Five leaves were assessed in each treatment group to determine chlorophyll content, and the average value was recorded as the SPAD value, in line with the methodology previously described [45]. To assess the relative water content (RWC), fresh leaves were submerged in Petri dishes filled with distilled water, and these dishes were subsequently kept in a dark environment for a period of 4 h. Following this, the turgid weight (TW) of the leaves was recorded. Subsequently, the leaves were subjected to oven drying to obtain the dry weight (DW). The RWC was then calculated using the formula: $RWC=\frac{FW-DW}{TW-DW}\times 100,$ as outlined in reference [46].

### 2.4. Electrolyte Leakage

To determine electrolyte leakage, 100 mg of freshly collected leaf samples were cut into 5 mm pieces and submerged in test tubes containing 10 mL of distilled deionized water. These tubes were sealed with plastic caps and positioned in a water bath maintained at a constant temperature of 32 °C. After a 2 h period, the initial electrical conductivity of the solution (EC1) was gauged using an electrical conductivity meter (CM-115, Kyoto Electronics, Kyoto, Japan). Subsequently, the leaf samples were autoclaved at 121 °C for 20 min to ensure the complete breakdown of tissues, allowing the release of all electrolytes. Following autoclaving, the samples were cooled to 25 °C, and the final electrical conductivity (EC2) was measured. The electrolyte leakage (EL) was then calculated using the formula $EL=\frac{EC1}{EC2}\times 100$, expressing the percentage of electrolytes released from the leaf tissues [45].

### 2.5. Measurement of H_2_O_2_, MDA, and Superoxide Anion (O_2_^−^) Contents

H_2_O_2_ was determined by using the procedure in [47]. In brief, 0.1 g of leaf sample was homogenized and extracted with 5 mL of a 0.1% trichloroacetic acid (TCA) solution. The extract was then centrifuged at 12,000× *g* for 15 min. Subsequently, 0.5 mL of the supernatant was collected and mixed with 1 mL of 1 M potassium iodide and 0.5 mL of 10 mM phosphate buffer (pH 7.0), and the absorbance was measured at 390 nm.

To assess lipid peroxidation in the leaves, malondialdehyde (MDA) levels were determined according to the method described by [48]. In brief, 0.1 g of fresh plant tissue was ground and mixed with 10 mL of 5% TCA. After centrifugation at 4000× *g* for 10 min at 4 °C, the resulting supernatant was combined with 4 mL of thiobarbituric acid (TBA) solution, heated at 90 °C for 25 min, and then rapidly cooled to 4 °C. The absorbance of the sample was measured at 532 and 600 nm, and the MDA content was calculated as µmol g^−1^ of fresh weight (FW).

Superoxide anion production was measured according to the method described by [49]. Fresh plant samples (200 mg) were homogenized in liquid nitrogen, and 100 mM sodium–phosphate buffer containing 1 mM diethyl dithiocarbamate was added. The resulting supernatant, obtained after centrifugation, was assessed for superoxide anion levels by its NBT-reducing capacity in a buffered solution. The absorbance was read at 540 nm.

### 2.6. Determination of Antioxidant Enzyme Activities

To quantify the CAT (catalase) enzyme activity, the previously described method [50] was used. The reaction was initiated with 15 mM of H_2_O_2_ and 50 mM of phosphate buffer (pH 7.0), then 100 μL of extract was added. Catalase activity was found by the decrease in absorbance of H_2_O_2_ at 240 nm, and CAT one unit was defined as micrograms of H_2_O_2_ released per milligram of protein per minute.

SOD (Superoxide dismutase)activity was assessed using the procedure of [46], which involved measuring SODs capacity to reduce nitroblue tetrazolium (NBT) under photochemical conditions. SOD activity units were quantified as the quantity of enzyme needed to make a 50% inhibition in NBT reduction, as observed at 560 nm.

For APX (Ascorbate peroxidase) activity measurement, 100 mg of plant sample underwent extraction using 1 mL of 50 mM phosphate buffer (pH 7.0) comprising 1 mM ascorbic acid and 1 mM EDTA. Following centrifugation of the homogenates at 4000× *g* (4 °C) for 15 min, the resulting supernatant was combined with a solution containing phosphate buffer (pH 7.0), 15 mM ascorbic acid, and 0.3 mM H_2_O_2_. Subsequently, the absorbance of the reaction mixture was measured at 290 nm [51].

To measure GSH (Glutathione) content, we used the method of [52] with some modifications and a method used in our pervious study [44]. Briefly, leaf samples were ground in liquid nitrogen and treated with 10% trichloroacetic acid. Following centrifugation, the resulting supernatant was mixed with NaH_2_PO_4_ and DTNB solutions, then incubated for 5 min at 30 °C. The glutathione concentration was determined by measuring absorbance at 412 nm against a standard curve. The experiment was repeated three times for accuracy.

### 2.7. RNA Isolation and rt-qPCR

Leaf samples were collected from each experimental group in triplicate after 48 h of exposure to stress. Total RNA extraction, cDNA synthesis, and quantitative real-time PCR (rt-qPCR) were performed following the protocols described by [53]. The gene primers utilized in this study were obtained from the NCBI GenBank database. To design these primers, Primer3 http://primer3.ut.ee (accessed on 3 June 2024) and NCBI Primer-BLAST software https://www.ncbi.nlm.nih.gov/ (accessed on 3 June 2024) tools were employed, adhering to stringent criteria such as primer length, GC content, and melting temperature. To ensure their specificity, each primer sequence underwent BLAST searches against the reference genome. The primer sequences and accession numbers for the gene analysis are presented in Table 1. Total RNA extraction was carried out using RNeasy Plant Mini Kits from Qiagen Hilden, Germany, while cDNA synthesis and rt-qPCR were conducted using qPCRBIO and qPCRBIO SYBR Green Kits from PCR Biosystems London, United Kingdom, respectively. Actin served as the reference gene [54]. For rt-qPCR, an Eco Real-Time machine (Illumina, Singapore) was employed, with a reaction volume of 20 μL, consisting of 10 μL of SYBR green, 7 μL of ddH_2_O, 1 μL of template DNA, and1 μL of forward and 1 μL of reverse primer. The delta-delta Ct (ΔΔCt) method was used for comparing gene expression levels between different experimental groups.

### 2.8. Determination of Cu^2+^ Ca^2+^, Mg^2+^, and K^+^ Contents

To analyze the mineral contents, samples of rice seedlings were carefully harvested and thoroughly washed with deionized water (dH_2_O) to eliminate surface impurities. Subsequently, the samples underwent oven-drying at 70 °C for complete drying and were then crushed in liquid nitrogen. For the determination of potassium ion (K^+^) content, flame photometry was employed, utilizing a Jencon PFP 7 flame photometer (JENCONS-PLS, Leighton Buzzard, UK) [55]. The calcium ion (Ca^2+^) and magnesium ion (Mg^2+^) contents were determined through complexometric titration utilizing ethylenediaminetetraacetic acid disodium salt, as described by [56,57]. To determine Cu^2+^ contents, plant samples were crushed into fine powder using liquid nitrogen. This powdered form was combined with a solution containing 7 mL of 65% HNO_3_ and 1 mL of 30% H_2_O_2_. The mixture underwent microwave treatment at 180 °C for 20 min before being allowed to cool for 40 min. The extract obtained from this process was subjected to analysis via ICP-MS (Optima 7900DV, Perkin-Elmer, Waltham, MA, USA) to quantify the concentrations of Cu^2+^ present in the plant.

### 2.9. Statistical Analysis

The experiments were conducted in three independent sets of experiments for each group of plants, and the data from each replicate were subsequently combined. Mean values, presented with a standard error, were analyzed for significant differences between treatments using one-way analysis of variance (ANOVA). Post hoc analysis was conducted using Duncan’s multiple range test, performed through SPSS version 29.0.0. The outcomes were graphically depicted using GraphPad Prism software (version 8.0, San Diego, CA, USA). Correlation between the studied traits was evaluated using Pearson’s correlation coefficient with Origin software (Version 2024, OriginLab Corporation Northampton, MA, USA).

## 3. Results

### 3.1. Effects of GABA Application on Rice Growth under Copper Stress

**Figure 1:**
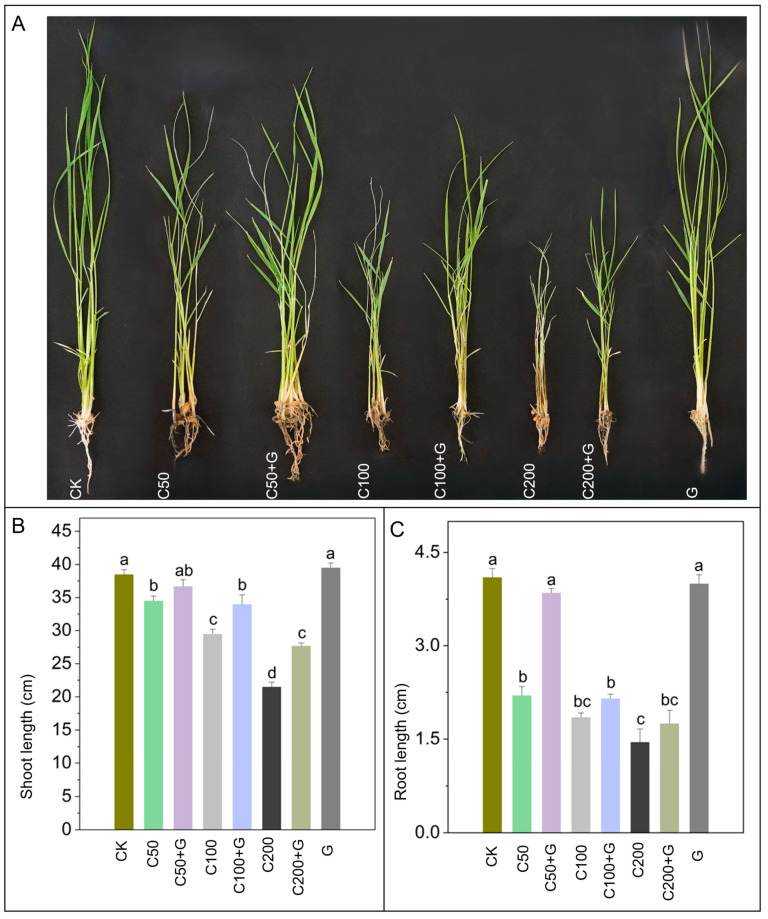
Evaluation of growth parameters in rice plants under normal conditions, copper stressed conditions and GABA supplemented copper stressed conditions. (**A**) shows effects of copper stress on overall plant growth, (**B**) shoot length, (**C**) shows root length. Whereas CK is control, C50 is 50 µM copper, C50+G is 50 µM copper and 1 mM GABA, C100 is 100 µM copper, C100+G is 100 µM copper and 1 mM GABA, C200 is 200 µM copper, C200+G is 200 µM copper and 1 mM GABA, and G is only 1 mM GABA treatment respectively. Each column represents the means of three replicates ±SE. Distinct lower case letters indicate statistically significant differences within each treatment group, as determined by Duncan’s multiple range test at a significance level of *p* ≤ 0.05.

The detrimental effects of copper stress on rice plant growth were evident, as indicated by a significant reduction in plant height across varying concentrations of copper (C50, C100, and C200) compared to control plants. Specifically, there was a decrease of 20%, 26%, and 42% in plant height for C50, C100, and C200, respectively. Similarly, root length also exhibited substantial reductions of 42%, 62%, and 65% in the C50, C100, and C200, respectively, compared to the control group. However, the introduction of GABA supplementation alongside copper stress demonstrated a positive impact on plant growth. While a non-significant increase in plant height was observed in the C50+G compared to C50, notable improvements were observed with GABA application in higher copper concentrations. Specifically, plant height increased by 18% in C100+G and 32% in C200+G compared to their respective stressed plants. Conversely, the effects on root length varied with GABA treatment. Significant enhancement in root length by 80% was observed in C50+G compared to C50, while no substantial increment was recorded in other treatments compared to their respective stressed conditions. Additionally, sole GABA application exhibited a marked increase in both shoot and root length compared to the stressed plants, as illustrated in Figure 1.

**Figure 2:**
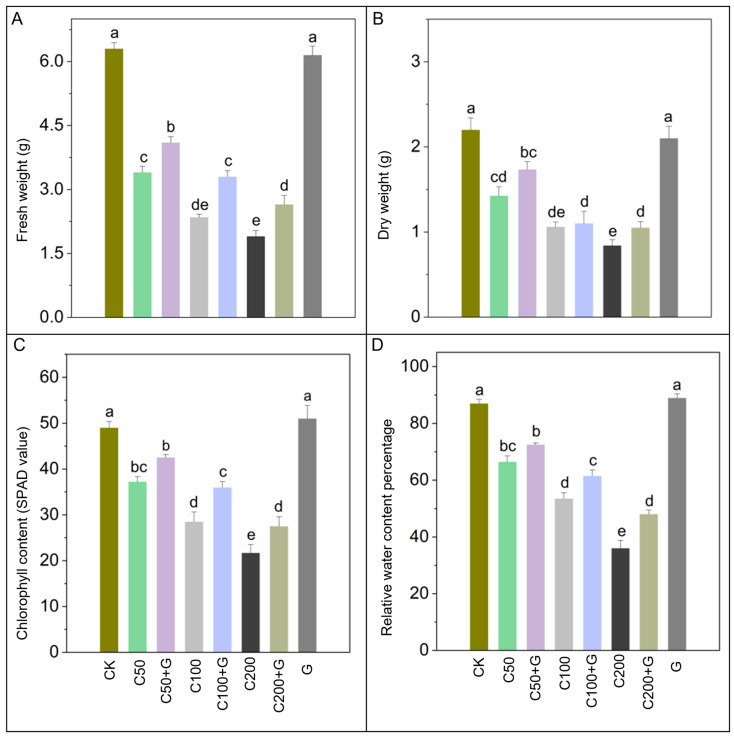
Effects of GABA on (**A**) fresh weight, (**B**) dry weight, (**C**) chlorophyll content, and (**D**) relative water content of rice plants under copper stress. Whereas CK is control, C50 is 50 µM copper, C50+G is 50 µM copper and 1 mM GABA, C100 is 100 µM copper, C100+G is 100 µM copper and 1 mM GABA, C200 is 200 µM copper, C200+G is 200 µM copper and 1 mM GABA, and G is only 1 mM GABA treatment respectively. Each column represents the means of three replicates ±SE. Distinct lower case letters indicate statistically significant differences within each treatment group, as determined by Duncan’s multiple range test at a significance level of *p* ≤ 0.05.

Furthermore, the detrimental impact of copper stress on plant biomass was apparent. Plant fresh weight declined by 48%, 67%, and 74%, while dry weight decreased by 31%, 50%, and 59% in C50, C100, and C200 treatments, respectively, compared to the control group. However, the application of GABA yielded significant improvements. Fresh weight increased by 28%, 52%, and 60% in C50+G, C100+G, and C200+G treatments, respectively, compared to their corresponding stressed plants. Moreover, dry weight exhibited a substantial increase in C200+G compared to C200 plants. Furthermore, plants treated solely with GABA also displayed notable enhancements in both fresh and dry weight compared to stressed plants, as depicted in Figure 2A,B.

### 3.2. Effects of GABA on Regulation of Chlorophyll Contents and Relative Water Contents

In this study, significant reductions were observed in both chlorophyll contents and relative water contents due to copper stress. Specifically, copper stress resulted in a substantial decrease of 23%, 43%, and 56% in chlorophyll contents and 22%, 41%, and 42% in relative water contents in the C50, C100, and C200 treatments, respectively, compared to control plants. However, the introduction of GABA demonstrated significant restorative effects, effectively mitigating these declines. Notably, GABA treatment led to increases of 30% and 24% in chlorophyll contents and 24% and 26% in relative water contents in the C100+G and C200+G groups, respectively, compared to their respective stressed groups. Conversely, C50+G did not exhibit a significant improvement in chlorophyll and relative water contents compared to C50. Furthermore, plants treated solely with GABA also displayed notable enhancements in both chlorophyll and relative water contents compared to stressed plants, as illustrated in Figure 2C,D.

### 3.3. Effects of GABA on Regulation of ROS and Electrolyte Leakage in Response to Copper Stress

**Figure 3:**
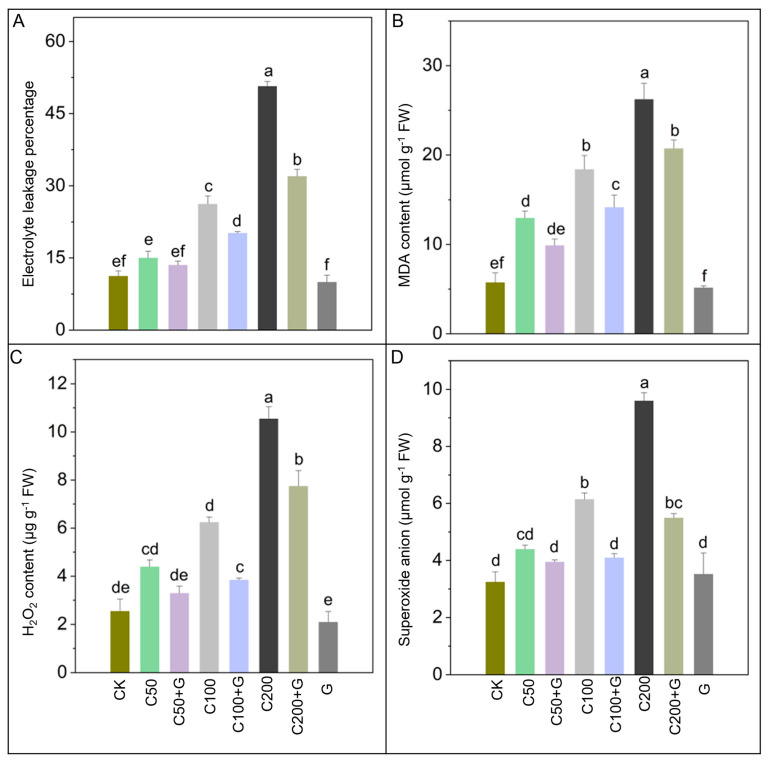
Effects of GABA on electrolyte leakage and reactive oxygen species of rice plants under copper stress. Where, (**A**) is electrolyte leakage, (**B**) is MDA content, (**C**) is H_2_O_2_ content, and (**D**) is superoxide anion. Whereas CK is control, C50 is 50 µM copper, C50+G is 50 µM copper and 1 mM GABA, C100 is 100 µM copper, C100+G is 100 µM copper and 1 mM GABA, C200 is 200 µM copper, C200+G is 200 µM copper and 1 mM GABA, and G is only 1 mM GABA treatment respectively. Each data bar represents means of three replicates ±SE. Distinct lower case letters indicate statistically significant differences within each treatment group, as determined by Duncan’s multiple range test at a significance level of *p* ≤ 0.05.

In the investigation into the impact of copper stress on rice plants, crucial indicators of oxidative stress, like electrolyte leakage, MDA (malondialdehyde) content, H_2_O_2_ (hydrogen peroxide) concentration, and O_2_^−^ (superoxide anion) production, were meticulously examined. The study revealed a significant escalation in electrolyte leakage by 129% and 308% in the C100 and C200 treatments compared with control plants, while electrolyte leakage increased non-significantly in the C50 group compared to the control. Similarly, MDA contents exhibited a substantial increase of 116%, 202%, and 150% in the C50, C100, and C200 treatments, respectively, compared to the control group. H_2_O_2_ contents showed a non-significant increase in the C50 treatment, while copper stress in the C100 and C200 groups led to a substantial increment of 200% and 267% compared to the control group. Moreover, superoxide anion production did not show a significant increase in the C50 group, while an 87% and 200% increase were recorded in the C100 and C200 groups compared with the control group. This surge strongly suggests the detrimental influence of reactive oxygen species (ROS) formation, causing considerable harm to cellular integrity. However, the external application of GABA yielded promising results, notably reducing oxidative stress parameters. Exogenous application of GABA had a non-significant impact on C50+G in all ROS parameters, while in the C100+G and C200+G groups, GABA decreased electrolyte leakage by 34% and 39%, MDA by 22% and 27%, H_2_O_2_ by 38% and 30%, and O_2_^−^ by 33% and 39% compared with their respective stressed groups, as depicted in the accompanying Figure 3. Furthermore, plants treated solely with GABA also displayed notable enhancements in these parameters compared to stressed plants. These findings underscore the potential of GABA in mitigating oxidative stress-induced damage in rice plants.

### 3.4. Effects of GABA on Regulation of Antioxidant Enzymes under Copper Stress

**Figure 4:**
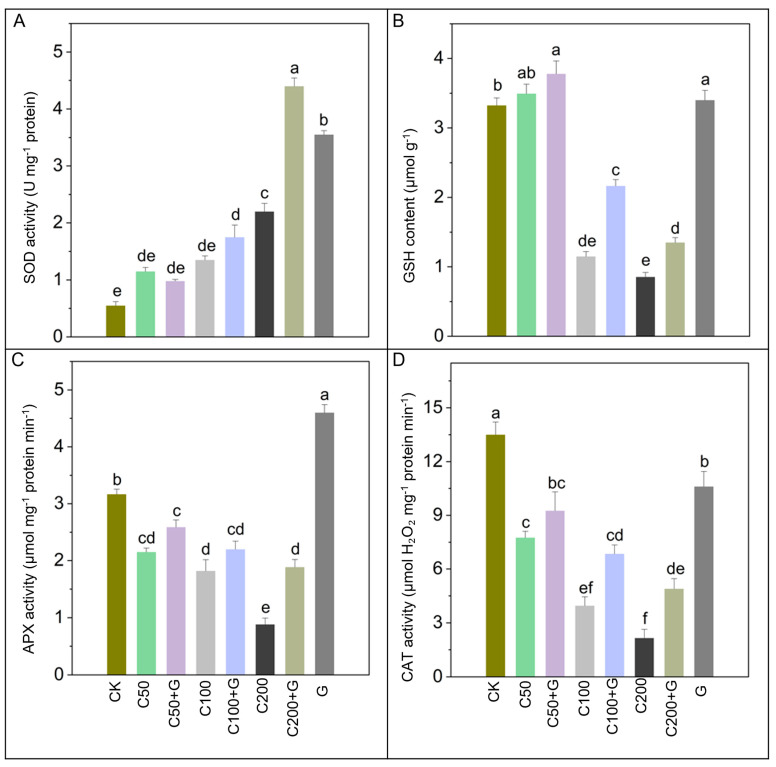
Effects of GABA on antioxidant enzymes activities of rice plants under copper stress. (**A**) shows (SOD), (**B**) GSH, (**C**) APX, and (**D**) CAT. Whereas CK is control, C50 is 50 µM copper, C50+G is 50 µM copper and 1 mM GABA, C100 is 100 µM copper, C100+G is 100 µM copper and 1 mM GABA, C200 is 200 µM copper, C200+G is 200 µM copper and 1 mM GABA, and G is only 1 mM GABA treatment respectively. Each data point represents the means of three replicates ±SE. Distinct lower case letters indicate statistically significant differences within each treatment group, as determined by Duncan’s multiple range test at a significance level of *p ≤* 0.05.

The investigation into antioxidant responses in rice plants subjected to copper stress, along with GABA supplementation, yielded significant findings. Antioxidant enzymes exhibited varying responses to copper stress, with notable alterations in these activities observed. Specifically, copper stress induced a gradual increase in SOD activity by 100%, 150%, and 266% in the C50, C100, and C200 treatments, respectively, compared to the control group. Conversely, GSH activity displayed a slight rise in the C50 group but significantly declined by 64% and 89% in the C100 and C200 groups, respectively, compared to the control group. Similarly, APX and CAT activities exhibited downward trends under copper stress, with APX decreasing by 34%, 43%, and 72%, and CAT decreasing by 42%, 69%, and 80% in the C50, C100, and C200 groups, respectively, compared to the control group. However, concurrent application of GABA led to significant enhancements in these enzyme activities. In the C100+G and C200+G groups, GABA application resulted in significant increments of 22% and 125% in SOD, 69% and 80% in GSH, and 75% and 100% in CAT compared to their respective stressed plants. Notably, GABA treatment did not elevate APX activity in the C50+G and C100+G groups, although a substantial 125% increase was recorded in the C200+G group compared to the respective stressed plants. Additionally, plants treated solely with GABA also exhibited notable enhancements in these enzymatic activities compared to stressed plants, as depicted in Figure 4.

### 3.5. Effects of GABA on Regulation of Cu^2+^, Ca^2+^, Mg^2+^, and K^+^ Regulation in Response to Copper Stress

**Figure 5:**
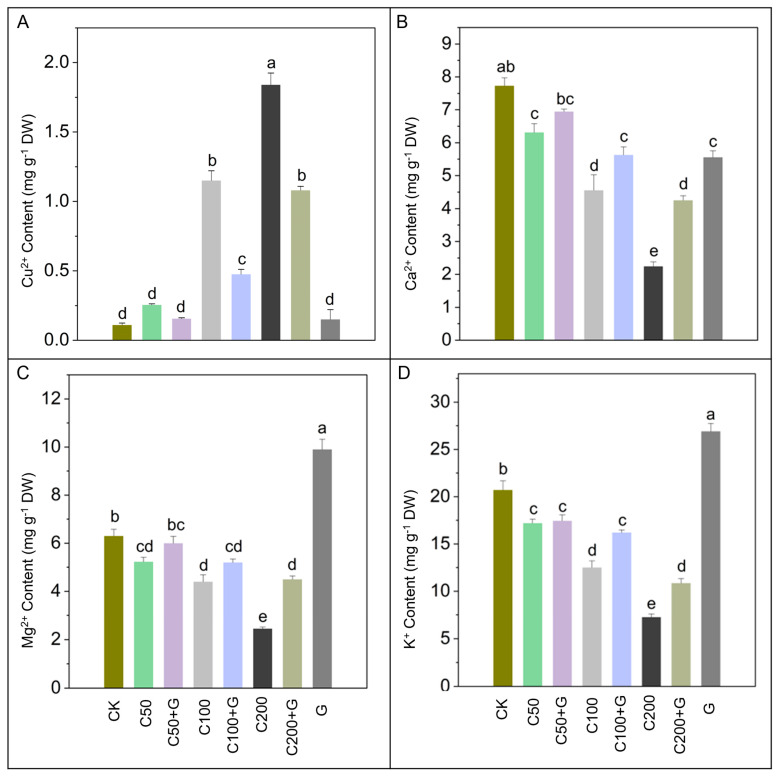
Effects of GABA on (**A**) copper, (**B**) calcium, (**C**) magnesium, and (**D**) potassium content accumulation in rice plants under copper stress. Whereas CK is control, C50 is 50 µM copper, C50+G is 50 µM copper and 1 mM GABA, C100 is 100 µM copper, C100+G is 100 µM copper and 1 mM GABA, C200 is 200 µM copper, C200+G is 200 µM copper and 1 mM GABA, and G is only 1 mM GABA treatment respectively. Each column represents the means of three replicates ±SE. Distinct lower case letters indicate statistically significant differences within each treatment group, as determined by Duncan’s multiple range test at a significance level of *p ≤* 0.05.

The impact of copper stress on mineral accumulation within rice plants was notably conspicuous, with a marked surge observed in Cu^2+^ levels by 1000% in the C100 and 1700% in the C200 groups compared with control plants, while the C50 plants exhibited a non-significant increment in comparison to the control plants. Conversely, the application of GABA under copper stress conditions demonstrated a remarkable effect on copper uptake, revealing a substantial reduction of 54% and 47% in the C100+G and C200+G groups, respectively, compared to their respective stressed groups. Moreover, copper stress significantly decreased the uptake of essential minerals, with reductions of 22%, 40%, and 69% observed in Ca^2+^, 19%, 32%, and 64% in Mg^2+^, and 26%, 42%, and 66% in K^+^ in the C50+G, C100+G, and C200+G groups, respectively, compared to their respective stress plants. However, the application of GABA led to non-significant increases in mineral accumulation in the C50+G groups compared to C50, while substantial increments of 26% and 82% in Ca^2+^, 28% and 128% in K^+^ were recorded in the C100+G and C200+G groups, respectively, compared to their respective stressed groups. Additionally, Mg^2+^ showed a significant increment in the C200+G group compared with C200, as depicted in Figure 5. These findings suggest the potential of GABA as a protective agent against excessive copper accumulation and for maintaining essential mineral homeostasis in rice plants.

### 3.6. Effects of GABA on Regulation of Gens Expression in Response to Copper Stress

**Figure 6:**
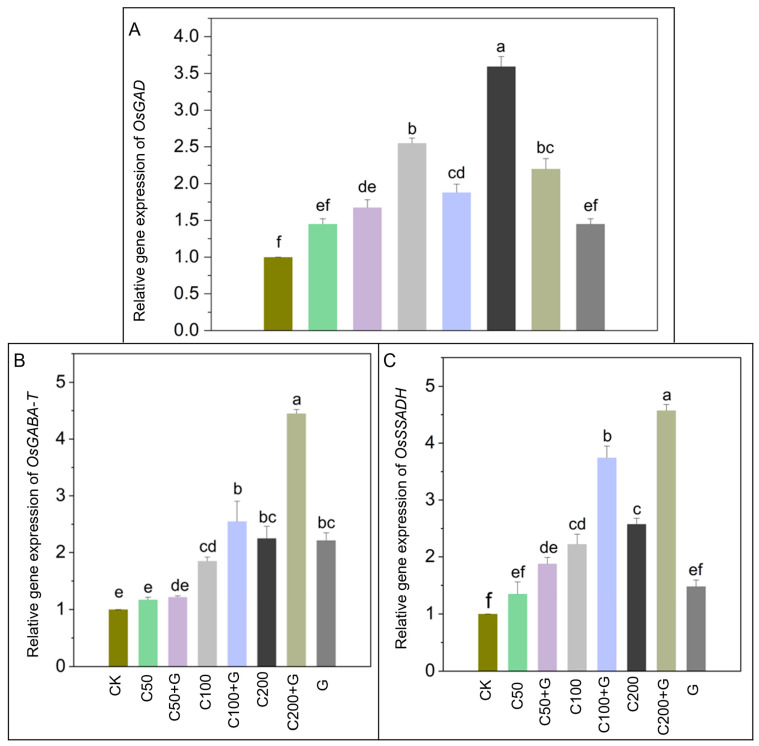
Effects of GABA on relative gene expression of (**A**) *OsGAD*, (**B**) *OsGABA-T*, and (**C**) *OsSSADH* in rice plants under copper stress. Actin was used as the reference gene. Whereas CK is control, C50 is 50 µM copper, C50+G is 50 µM copper and 1 mM GABA, C100 is 100 µM copper, C100+G is 100 µM copper and 1 mM GABA, C200 is 200 µM copper, C200+G is 200 µM copper and 1 mM GABA, and G is only 1 mM GABA treatment respectively. Each column represents the means of three replicates ± SE. Distinct lower case letters indicate statistically significant differences within each treatment group, as determined by Duncan’s multiple range test at a significance level of *p ≤* 0.05.

The examination of GABA’s impact on gene expression during copper stress revealed significant and diverse effects on GABA shunt genes. Under copper stress, glutamate decarboxylase (*OsGAD*) exhibited a 127% and 230% increase in expression in the C100 and C200 groups, while the C50 group showed a non-significant increase compared to the control group. However, the introduction of GABA during copper stress caused a slight non-significant increase in the C50+G group, while the C100+G and C200+G groups showed a substantial 32% and 47% decrease in *OsGAD* gene expression, respectively, compared to their respective stressed groups, as shown in Figure 6A. This implies a pronounced regulatory effect, potentially linked to the downregulation of GABA biosynthesis. Conversely, the gene associated with GABA transaminase (*OsGABA-T*) displayed a 72% and 127% increase in the C100 and C200 groups under copper stress compared to controls. Yet, with GABA application during copper stress, there was a remarkable 38% and 80% elevation in *OsGABA-T* gene expression in the C100+G and C200+G groups compared to their respective stressed plants, highlighting the pivotal role of GABA in modulating gene expression. Moreover, succinic semialdehyde dehydrogenase (*OsSSADH*) exhibited a substantial 76% and 107% increase in expression in the C100 and C200 groups under copper stress compared to controls. Impressively, GABA supplementation during copper stress resulted in a notable 60% and 94% increase in *OsSSADH* gene expression in the C100+G and C200+G groups, respectively, emphasizing GABA’s potential in regulating the GABA shunt pathway under stress conditions. However, the C50+G group did not show a significant increase in OsGABA-T and *OsSSADH* compared to their respective stressed plants, as depicted in Figure 6B,C.

### 3.7. Correlation between Plant Growth, Antioxidant Enzymes Activity, and Gene Expression

**Figure 7:**
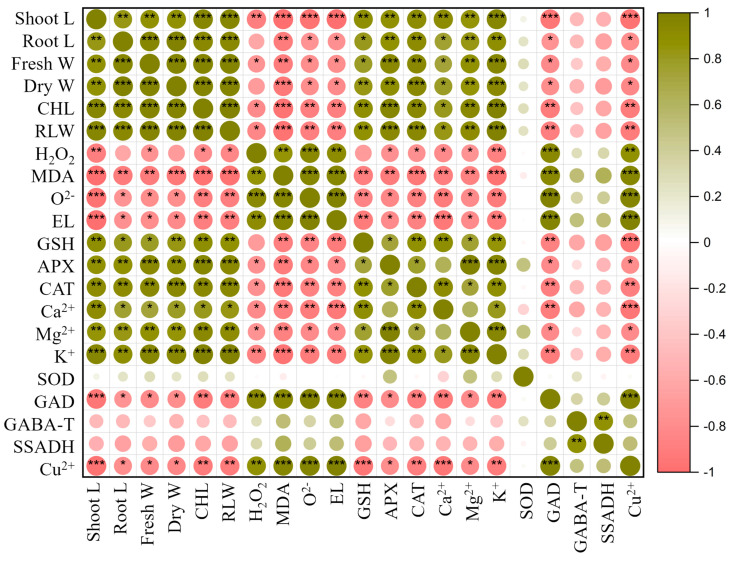
Pearson’s correlation matrix between rice growth attributes, reactive oxygen species, anti-oxidant enzymes, and gene expression in copper-stressed plants. Correlations are displayed in green (positive) and red (negative); color intensity and circle size are proportional to the correlation coefficient. * indicates *p* < 0.05, ** indicates *p* < 0.01, and *** indicates *p* < 0.001.

In this investigation, we examined the interrelationships among various growth characteristics, antioxidant enzyme activity, and gene expression in rice plants. Through Pearson correlation analysis, as depicted in Figure 7, we identified positive associations among several growth attributes, including shoot length, root length, plant biomass, chlorophyll index, relative water content, antioxidant enzymes, and mineral content concentration. These findings suggest a cohesive linkage among these attributes, indicating their mutual influence on developmental processes. Conversely, these attributes exhibited negative correlations with H_2_O_2_, O^2−^, MDA, electrolyte leakage, and copper content, implying that the accumulation of these substances may impede overall plant growth. Regarding gene expression, we observed a positive correlation between *OsGABA-T* and *OsSSADH* genes with H_2_O_2_, O^2−^, MDA, electrolyte leakage, and copper content. Similarly, the *OsGAD* gene displayed a robust positive correlation with these parameters. Notably, gene expression did not exhibit significant correlations with morphological parameters. Furthermore, heightened copper content demonstrated a strong positive correlation with H_2_O_2_, O^2−^, MDA, electrolyte leakage, and a strong negative correlation with Ca, Mg, and K contents, suggesting that elevated copper levels may exacerbate reactive oxygen species production during stressful conditions and impede the uptake of essential mineral nutrients.

## 4. Discussion

Copper (Cu) is an indispensable element crucial for optimal plant growth, actively engaged in diverse physiological processes such as photosynthesis, respiration, carbohydrate transport, nitrogen reduction and fixation, and protein metabolism [16]. Nevertheless, elevated levels of copper can induce toxicity in plants [58]. The investigation into the effects of elevated copper (Cu), specifically the moderate 100 µM and high 200 µM concentrations, on the growth parameters of rice plants has unveiled a notable reduction in these growth indicators, providing clear evidence of Cu-induced toxicity. Specifically, copper exerts significant impacts on key growth parameters, including shoot length, root length, fresh weight, and dry weight as visually depicted in Figure 1. These findings are in alignment with earlier studies conducted in rice by [59,60,61,62] and in (Shiny Elsholtzia) *Elsholtzia splendens* by [63] under conditions of copper-induced stress. The decline in growth parameters may be attributed to decreased cell expansion [64], impaired nutrient uptake [16], and alteration of the photosynthetic machinery caused by copper-induced stress [65]. Gamma-aminobutyric acid (GABA) plays an essential role in various plant processes, particularly in enhancing plant resilience to a wide range of abiotic and biotic stress conditions. GABA acts as a key signaling molecule that promotes the growth and development of rice plants under abiotic stress conditions [66]. The findings from this study clearly demonstrate that the exogenous application of GABA has a substantial positive effect on mitigating the detrimental impacts of copper stress on rice plant morphology, as shown in Figure 1. Similar findings were reported by [30,40], indicating that GABA alleviated the inhibitory effects on growth, resulting in increased plant height, root length, fresh weight, and dry weight of barley and mustard seedlings during aluminum and chromium-induced stress. This improvement could be attributed to its positive effects on factors such as water status, photosynthetic activity, and the up-regulation of antioxidant enzymes.

The external application of GABA presents a promising strategy for ameliorating copper-induced moderate and severe stress in rice plants, as evidenced by improvements in leaf water retention capacity and chlorophyll levels Figure 2C,D. The decline in relative water content and leaf chlorophyll content under copper stress reflects the onset of oxidative stress, characterized by heightened chlorophyllase activity and photo-oxidation of reactive oxygen species [67,68]. This oxidative stress-induced chlorophyll degradation adversely impacts photosynthetic efficiency, gas exchange processes, and ultimately, the growth and development of rice plants [69]. GABA offered partial protection to the photosynthetic pigments by scavenging deleterious ROS activities through mechanisms involving proline accumulation and activation of the antioxidative defense system [70]. GABA plays a crucial role in activating the antioxidative defense system, including enzymes such as superoxide dismutase, catalase, and peroxidase, which collectively detoxify ROS and alleviate oxidative stress [71]. GABA modulates these interconnected physiological mechanisms to enhance rice performance under Cu-induced stress.

Results indicated a significant increase in H_2_O_2_, O_2_^−^, MDA, and electrolyte leakage due to moderate and high Cu exposure in rice plants, as shown in Figure 3. While plants naturally generate reactive oxygen species (ROS) as signaling molecules in response to various stresses, our observations suggest that under Cu stress conditions, the accelerated production of ROS leads to the oxidation and [72] degradation of crucial cellular structures, which align with the results of [73], thereby disrupting normal cellular functionality. Elevated levels of MDA indicate increased lipid peroxidation and electrolyte leakage, reflecting compromised membrane integrity [74]. Additionally, higher concentrations of H_2_O_2_, a potent ROS, can react with both micro- and macro-biological molecules, potentially altering their structures and functions. In this study, copper stress leads to an increase in electrolyte leakage and the generation of reactive oxygen species (ROS) such as O_2_^−^, H_2_O_2_, and OH, consequently causing oxidative damage and eventual cell death in plants, as supported by the findings of Chen et al., Ahsan, et al., and Jonak, et al. [61,75,76]. Observing plants treated with GABA revealed a noticeable decrease in oxidative stress compared to untreated plants. This strongly indicates the potential of GABA in scavenging ROS, consequently minimizing oxidative damage in rice plants subjected to Cu toxicity. GABA likely plays a role in scavenging reactive oxygen species (ROS) and potentially regulating various processes in plants [77]. The GABA shunt pathway, which supplies succinate and nicotinamide adenine dinucleotide (NADH) during respiration, suggests that GABA regulation in plants could directly impact the management of ROS, particularly under conditions of oxidative stress [78]. Previous studies have also noted a decrease in oxidative stress in plants treated with GABA under conditions of metal toxicity [40,41].

The results of our study indicate that rice plants exhibited an increase in SOD, GSH, APX, and CAT activities in response to Cu-induced stress. The rise in GSH content observed at the initial stages, followed by a decrease as copper concentration increased, as depicted in Figure 4 is likely indicative of plant antioxidant defense mechanisms. This initial increase can be interpreted as a preemptive response aimed at mitigating potential oxidative damage [72]. It may also reflect an upregulation of enzymes responsible for GSH synthesis [79], and the activation of redox-sensitive signaling pathways [80]. Previous findings have reported similar observations, showing that Cu-induced plants exhibit increased antioxidant activities in rice [59] and in *Elsholtzia haichowensis* [81]. The increase in antioxidant activities observed in rice plants exposed to Cu stress suggests their potential roles in the adaptation process against Cu-induced oxidative stress. Nevertheless, external application of GABA significantly boosted the activities of SOD, GSH, APX, and CAT in rice plants subjected to copper stress (Figure 4). SOD and CAT play crucial roles in neutralizing reactive oxygen species. For instance, SOD detoxifies superoxide anions into O_2_^−^ and subsequently H_2_O_2_, which is further decomposed by CAT and POD enzymes [82]. Meanwhile, APX and GSH participate in the ascorbate-glutathione cycle, vital for detoxifying the harmful effects of ROS [83]. The difference between the lowered antioxidant activities and the high levels of ROS seen in GABA-lacking, copper-stressed plants suggest an imbalance in the production of reactive oxygen species (ROS) and the ability of antioxidant enzymes to neutralize them. The introduction of exogenous GABA mitigated this imbalance by boosting antioxidant responses, subsequently reducing ROS production, and enhancing the scavenging capability of antioxidant enzymes [84]. Our research corresponds with previous findings [85] that illustrate noteworthy adjustments in antioxidant enzymes, contributing to the removal of reactive oxygen species (ROS) in rice plants through the application of GABA.

Copper (Cu) stress can exert a significant influence on the concentration of essential nutrients such as calcium (Ca), magnesium (Mg), and potassium (K) in rice plants [86]. Elevated levels of copper in the soil can lead to alterations in root morphology, physiology, and nutrient transport mechanisms [87]. As a result, the uptake of nutrients such as Ca, Mg, and K may be impaired under copper stress conditions [88]. However, exogenous GABA can play a vital role in the survival of rice plants under copper stress conditions. Our findings illustrate that moderate and high copper stress in rice plants reduces the uptake of essential nutrients like Ca, Mg, and K, while GABA application counteract the impacts of copper on the uptake and accumulation of these nutrients, as depicted in Figure 5B–D. However, the application of GABA in the absence of stress conditions resulted in a decreased calcium content, as illustrated in Figure 5B. This reduction may be attributed to the inhibition of calcium channels, which subsequently reduces calcium influx into the cells [89]. This mechanism serves to prevent excessive intracellular calcium levels, which could otherwise be toxic to the cells [90]. Recent studies suggest that GABA may enhance the uptake of calcium [91], magnesium [92], and potassium [93] during metal stress. GABA application to different plants like mustard [30], rice [41], tobacco [35], and maize [84] increases these minerals uptake and transport during metal stress conditions, thereby potentially mitigating the detrimental effects of metal toxicity on plant physiology and overall growth.

Moreover, the absorption of Cu in rice plants is significantly influenced by various plant and soil-related factors such as soil pH [94], soil organic matter [95], and copper concentration [60,96]. Yet, the reduced Cu content in GABA-treated plants might stem from GABA’s protective role in preserving cell integrity, potentially resulting in lesser Cu uptake by the plants. Furthermore, the signaling role of GABA may contribute to the plants’ effective response to Cu stress. Our study revealed a notable increase in copper accumulation in rice plants under copper stress, while external application of GABA resulted in a significant decrease in copper accumulation (Figure 5A). In rice, ref. [33] reported analogous results, observing that the external application of GABA inhibited the accumulation of As by modulating gene expression linked to the GABA shunt. Additionally, ref. [30] demonstrated a similar trend in mustard seedlings, where GABA application led to a reduction in Cr uptake in both roots and shoots. The decline in copper accumulation could be attributed to the formation of phytochelatins resulting from the transformation of glutathione (GSH) [97,98], and phytochelatins play a significant role in plants’ resistance to metal poisoning [99].

The GABA shunt, a metabolic pathway crucial in regulating cellular metabolism, is of particular significance in plants, where it interfaces with various stress responses and developmental processes [100]. *GAD*, a prominent enzyme within this pathway, initiates the irreversible conversion of glutamate into GABA [101]. This metabolic route extends its reach into mitochondria, where it intertwines with the TCA cycle. Within this cellular powerhouse, GABA undergoes transformation into succinic semialdehyde (SSA) catalyzed by *GABA-T*, followed by oxidation by *SSADH*, ultimately yielding succinate [102]. Our study closely aligns with previous research by Wang et al. [103] and Cao et al. [104], which has demonstrated the impact of exogenously applied GABA on plant responses to abiotic stress. Specifically, our findings show a significant reduction in *GAD* expression under copper (Cu) stress conditions (Figure 6A). The observed reduction in *GAD* activity under copper stress conditions hints at the existence of a potential feedback inhibition loop involving GABA and *GAD* expression [105]. This phenomenon may be indicative of regulatory mechanisms through which elevated GABA levels could influence *GAD* activity in response to stressors [106]. Additionally, this regulatory process aligns with principles of energy conservation [107] and redox balance strategies [108], suggesting an intricate interplay between metabolic regulation and stress response mechanisms in plants.

Interestingly, the application of exogenous GABA during copper stress in rice plants led to an increase in the expression of *GABA-T* and *SSADH* (Figure 6B,C). This observed increment aligns with findings in muskmelon, where, under calcium nitrate stress, exogenous GABA elevated *GABA-T* activity [109]. Similarly, research by Kumar et al. [33] highlighted that supplementing with GABA significantly enhanced the expression of *GABA T2* and *SSADH*, which contributed to plant tolerance against As(III) toxicity. Additionally, it has been reported that *GABA-T* and *SSADH* play a crucial role in preventing the accumulation of reactive oxygen species (ROS) [104,110]. *GABA-T* and *SSADH* regulation suggest that GABA application might have a beneficial impact on mitigating copper stress damage in rice plants by ensuring the smooth functioning of the GABA shunt and TCA metabolism under copper-induced stress.

## 5. Conclusions

**Figure 8:**
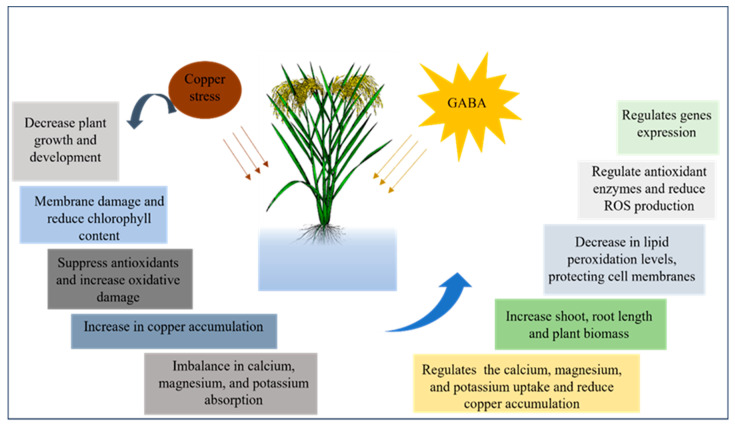
Summarizing the effects of copper stress on rice plants and the mitigating impact of GABA application highlights that GABA can alleviate copper-induced damage, promoting recovery and enhancing plant resilience.

The study highlights copper stress’s adverse effects on rice plants, impacting growth and increasing oxidative damage (Figure 8). However, GABA application effectively countered copper-induced stress. GABA supplementation reversed growth reduction, improved chlorophyll levels, and reduced ROS accumulation, reducing copper absorption and enhancing mineral uptake. GABA’s ability to strengthen antioxidant enzymes and regulate gene expression, especially in the GABA shunt pathway, played a crucial role in mitigating copper stress. Overall, GABA emerges as a promising solution, enhancing plant resilience and defending against oxidative damage during copper stress in rice plants. Utilizing GABA as a protective agent holds significant promise for transforming agricultural practices, providing a sustainable remedy to enhance plant resilience against environmental stressors. Further exploration and field experiments will be crucial in fully comprehending GABA’s broader applications and optimizing its utilization in shielding crops from oxidative damage due to copper stress.

## Figures and Tables

**Table 1:** Primers and accession numbers of selected genes designed through NCBI for rt-qPCR.

Gene	Forward Primers	Reverse Primers	Accession No
*OsActin*	CTGCGGGTATCCATGAGACT	GGAGCAAGGCAGTGATCTTC	X16280.1
*OsGAD*	TCGACATGGACGAGTACCCT	TGCCAGTCCTGCAAGCATAA	AK121088
*OsGABA-T*	AGGGTGGCTGAGCTGAAATC	TCACCGGTTCTGGGTGAATG	AK102306
*OsSSADH*	TTGGTCCAGTTGCACCACTT	AGCTCCAGGTACTCGTCCAT	AK060831

## Data Availability

The data presented in this study are available on request from the corresponding author.

## Supplementary Materials

The following supporting information can be downloaded at: https://www.mdpi.com/article/10.3390/antiox13060700/s1, Figure S1: Comparison of the effects of different concentrations of GABA on rice seedling, 1 mM treatment showed the highest root and shoot length as compared to control and 5 mM and 10 mM concentrations. Figure S2: 1 mM treatment of GABA application showed the highest root and shoot length (A) and highest fresh and dry weight (B) as compared to control and 5 mM and 10 mM concentrations. Each column represents the means of three replicates ±SE. Distinct lower case letters indicate statistically significant differences within each treatment group, as determined by Duncan’s multiple range test at a significance level of *p* ≤ 0.05. On the basis of these screening, we selected 1 mM of GABA.
