# Supplementary material for: Exogenous GABA Enhances Copper Stress Resilience in Rice Plants via Antioxidant Defense Mechanisms, Gene Regulation, Mineral Uptake, and Copper Homeostasis
Source: Antioxidants (Basel). 2024 Jun 7;13(6):700. doi: 10.3390/antiox13060700 (PMC11200589; doi:10.3390/antiox13060700)
Supplement: Supplementary file 1 [file antioxidants-13-00700-s001.zip › antioxidants-3027048-supplementary.pdf]

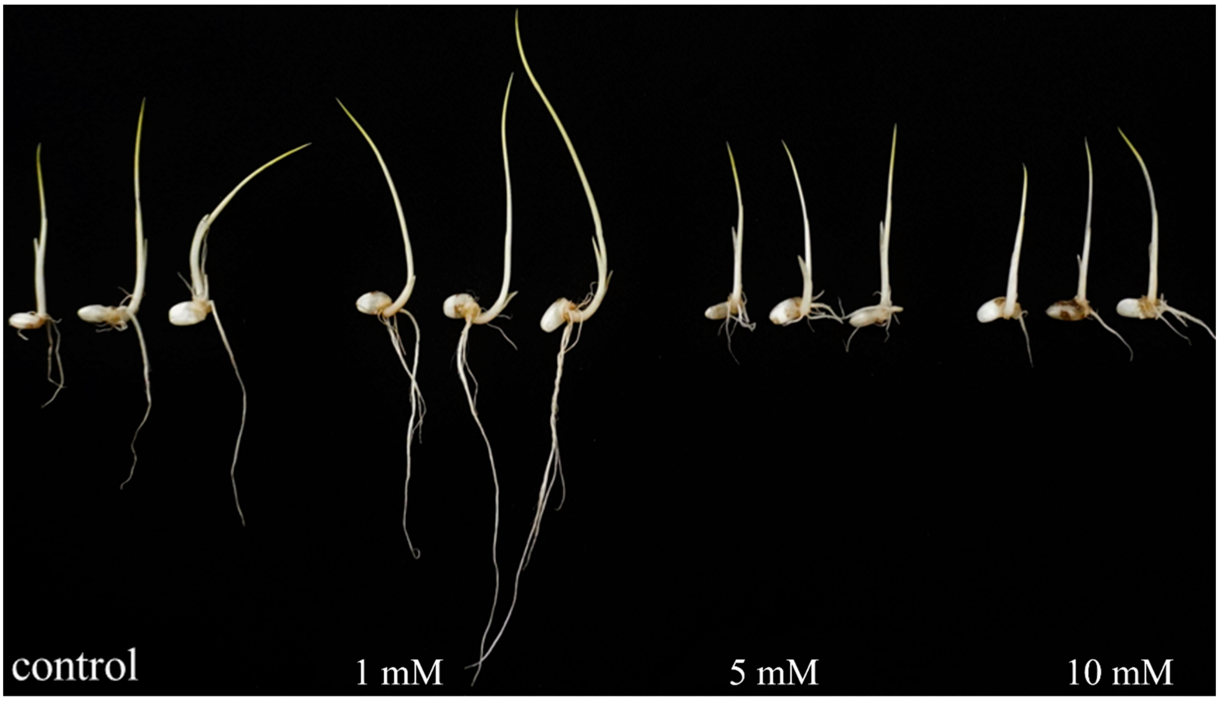

Supplementary Figure S1. Comparison of the effects of different concentrations of GABA on rice seedling, 1 mM treatment showed the highest root and shoot length as compared to control and 5 mM and 10 mM concentrations.

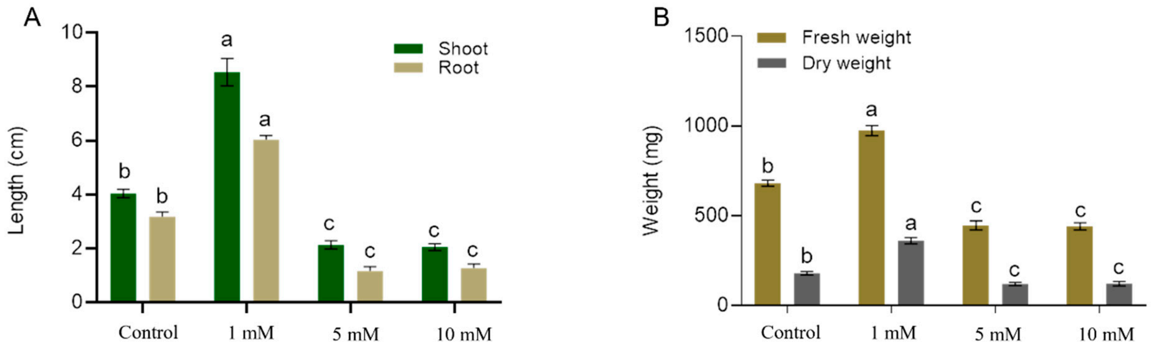

Supplementary Figure S2. 1 mM treatment of GABA application showed the highest root and shoot length (A) and highest fresh and dry weight (B) as compared to control and 5 mM and 10 mM concentrations. Each column represents the means of three replicates ± SE. Distinct lower case letters indicate statistically significant differences within each treatment group, as determined by Duncan's multiple range test at a significance level of $p \leq 0.05$. On the basis of these screening, we selected 1 mM of GABA.
